# Supplementary material for: Genome-wide CRISPR Screens in T Helper Cells Reveal Pervasive Crosstalk between Activation and Differentiation
Source: Cell. 2019 Feb 7;176(4):882–896.e18. doi: 10.1016/j.cell.2018.11.044 (PMC6370901; doi:10.1016/j.cell.2018.11.044)
Supplement: Data S2. Processed Data from All the Steps of the Analysis, Related to Figure 1 [file mmc2.zip › supplemental data/motif analysis/Stat6_SRX021632_homer/homerResults/motif2.similar.html]

motif2

## Information for motif2

C
A
T
G
G
T
A
C
A
G
T
C
G
T
A
C
A
G
T
C
A
G
T
C
G
C
A
T
C
T
A
G
A
T
C
G
G
C
A
T
A
C
T
G
C
A
T
G
  
Reverse Opposite:  

G
T
A
C
A
G
T
C
C
G
T
A
A
T
G
C
G
A
T
C
C
G
T
A
A
C
T
G
C
T
A
G
C
A
T
G
A
C
T
G
A
C
T
G
G
T
A
C
  

|  |  |
| --- | --- |
| p-value: | 1e-1585 |
| log p-value: | -3.650e+03 |
| Information Content per bp: | 1.746 |
| Number of Target Sequences with motif | 2173.0 |
| Percentage of Target Sequences with motif | 8.60% |
| Number of Background Sequences with motif | 160.2 |
| Percentage of Background Sequences with motif | 0.66% |
| Average Position of motif in Targets | 101.7 +/- 34.0bp |
| Average Position of motif in Background | 92.6 +/- 55.1bp |
| Strand Bias (log2 ratio + to - strand density) | 0.1 |
| Multiplicity (# of sites on avg that occur together) | 1.03 |
| Motif File: | file (matrix) reverse opposite |

### Similar de novo motifs found

|  |  |  |  |  |  |  |  |
| --- | --- | --- | --- | --- | --- | --- | --- |
| Rank | Match Score | Redundant Motif | P-value | log P-value | % of Targets | % of Background | Motif file |
| 1 | 0.903 | T A G C C T G A T A G C G A C T T G C A C T A G C T A G C A T G C A T G T A C G G T A C C T A G | 1e-1061 | -2443.061243 | 17.24% | 5.11% | motif file (matrix) |
| 2 | 0.907 | T G A C T G A C T C G A A T G C A G C T C G T A A C T G C T A G A C T G A T C G | 1e-997 | -2295.745293 | 10.41% | 2.02% | motif file (matrix) |
| 3 | 0.672 | C T G A A T C G A G T C A C T G A G T C A G T C T G A C A G C T A G T C A C G T | 1e-569 | -1311.211369 | 9.11% | 2.59% | motif file (matrix) |
| 4 | 0.711 | G C T A A C T G C A T G C A T G A C T G T C A G A G T C C T A G | 1e-475 | -1094.665301 | 16.07% | 7.32% | motif file (matrix) |
| 5 | 0.692 | C T A G A G T C T G A C C G T A A G T C A C G T C G T A A C T G | 1e-129 | -297.466012 | 3.05% | 1.11% | motif file (matrix) |
